# Primary adenocarcinoma of the stomach in von Recklinghausen's disease with high serum levels of multiple tumor markers: a case report

**DOI:** 10.1186/1752-1947-5-521

**Published:** 2011-10-23

**Authors:** Kazuya Kato, Atsushi Nagase, Kazuhiko Onodera, Minoru Matsuda, Yoshiaki Iwasaki, Yurina Kato, Kimitaka Kato, Takako Kawakami, Masahiko Taniguchi, Hiroyuki Furukawa

**Affiliations:** 1 Department of Surgery, Pippu Clinic, 2-10, 1 Cyome Nakamachi, Pippu Town Kamikawa-gun, Hokkaido, 078-0343, Japan; 2 Department of Surgery, Asahikawa Medical Center, 4048, 7 Cyome Hanasaki-cyou, Asahikawa, 070-8644, Japan; 3 Department of Surgery, Hokuyu Hospital, 5-1, 6-6 Higashi-Sappro, Shiroishi-ku Sapporo, 003-0006, Japan; 4 Department of Surgery, Nihon University, 1-8-13 Surugadai Kanda, Chiyoda-ku Tokyo, 010-8309, Japan; 5 Department of Digestive Internal Medicine, Okayama University, 2-5-1 Shikata Town, Okayama City, Okayama, 700-8558, Japan; 6 Department of Surgery, Asahikwa Medical College, 1-1, 2-1 Midorigaoka, Asahikawa, 078-8510, Japan

## Abstract

**Introduction:**

Gastric tumors in patients affected by neurofibromatosis type 1 are usually carcinoids or stromal tumors, and rarely adenocarcinomas.

**Case presentation:**

We report a case of an adenocarcinoma of the stomach in a 53-year-old Japanese man with neurofibromatosis type 1. An abdominal computed tomography scan and ultrasonography showed tumors in his liver. Gastric fibroscopy revealed a Borrmann type III tumor on his cardia that had spread to his esophagus and was highly suspicious for malignancy. Multiple biopsies showed an adenocarcinoma of the stomach, which was evaluated as gastric cancer, stage IV. Chemotherapy with TS-1 was performed. Our patient died four weeks after initial admission. Histological examination of a liver needle biopsy showed metastatic adenocarcinoma in his liver.

**Conclusion:**

To the best of our knowledge, high serum levels of α-fetoprotein, carcinoembryonic antigen, and carbohydrate antigen 72-4, resulting from gastric adenocarcinoma, have not been reported previously in a patient with neurofibromatosis type 1. We report this rare case along with a review of the literature.

## Introduction

Neurofibromatosis type 1 (NF-1), or von Recklinghausen's disease, is an autosomal dominant disorder characterized by cutaneous hyperpigmentation and multiple neurofibromas. The symptoms are café-au-lait spots, cutaneous neurofibromas and neoplasms of the peripheral or central nervous system. The genetic basis of the disease is a mutation on chromosome 17q11.2 [1]; however, no frequently recurring mutation has been identified. Malignancies are found in 3% to 15% of patients [2]. Occasionally reported in primary neoplasms, malignancies are most often associated with the peripheral or central nervous system. There have been reports of primary epithelial tumors of the gastrointestinal (GI) tract such as esophageal, gastric, small intestinal and colonic tumors [1,3], but GI involvement is rare [2]. We report a rare case of advanced gastric cancer in a patient with NF-1.

## Case presentation

**Figure 1:**
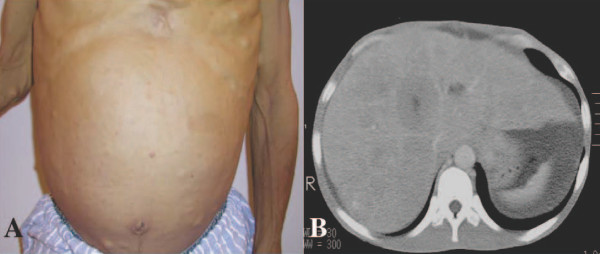
**Examinations on admission**. **(A) **Multiple discrete cutaneous neurofibromas and café au lait spots located on his abdominal wall. The abdominal wall was swollen with ascites. **(B) **An abdominal CT study showed multiple liver lesions and ascites.

**Figure 2:**
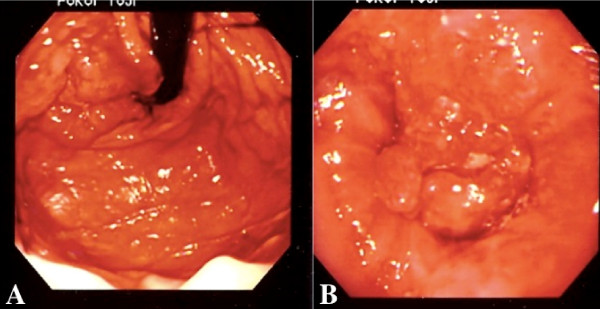
**Gastroendoscopic examination**. **(A) **A tumor was revealed on the cardia and extending to **(B) **his esophagus.

**Figure 3:**
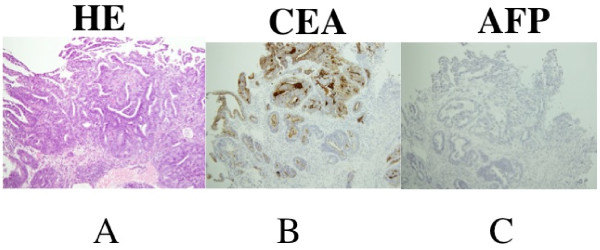
**Immunohistochemical study**. **(A) **Multiple biopsies showed a moderately differentiated tubular adenocarcinoma (×100). **(B) **Immunohistochemical determination of CEA in the area of the adenocarcinoma was positive (×100). **(C) **Immunohistochemical determination of AFP in the area of the adenocarcinoma was negative (×100).

**Figure 4:**
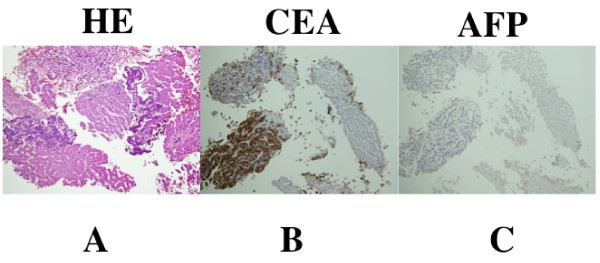
**Pathological review**. **(A) **Necropsy specimens of the liver showed moderately differentiated tubular adenocarcinoma (×100). **(B) **Immunohistochemical determination of CEA was positive (×100). **(C) **Immunohistochemical determination of AFP was negative (×100).

A 53-year-old Japanese man affected by NF-1 presented with a three-week history of jaundice, upper abdominal discomfort, dysphagia and loss of appetite (Figure 1A). His mother had a history of neurofibromatosis. Upon physical examination, a smooth mass, with its largest dimension measuring 20 cm, was palpated in his right upper abdomen. On admission, laboratory findings revealed leukocytosis, with a white blood cell count of 12,200/mm^3^; aspartate aminotransferase, 75 U/L; alanine aminotransferase, 75 U/L; alkaline phosphates 1913 U/L; γ-glutamyl transferase, 960 U/L; total protein, 7.4 g/dL; and total bilirubin, 4.4 mg/dL. His C-reactive protein level was 9.3 mg/mL (normal range, 0.5 mg/mL to 0.8 mg/mL). His serum level of carcinoembryonic antigen (CEA) was extremely high at 3050 ng/mL (cutoff, 2.5 ng/mL), and his α-fetoprotein (AFP) level was 812 ng/mL (cutoff, 10 ng/mL). The carbohydrate antigen (CA) 72-4 was also high at 180 U/mL (cutoff, 8.0 U/mL); CA 19-9 was normal at 16 U/mL (cutoff, 37 U/mL). An upper GI barium study showed a 5.0 cm filling defect on his cardia that extended to his lower esophagus. An abdominal computerized tomography (CT) scan showed multiple liver lesions and ascites, but no lymph node enlargement was identified (Figure 1B). Gastroendoscopic examination revealed a tumor with a 6 cm diameter on the esophagogastric junction, which was spreading to his esophagus (Figures 2A and 2B). Multiple biopsies showed moderately differentiated tubular adenocarcinoma of the stomach at stage IV (Figure 3A). An immunohistochemical study showed that CEA-positive and AFP-negative cells were present in the tumor (Figures 3B and 3C). Our patient was administered palliative chemotherapy and treated with TS-1 (tegafur, gimeracil, oteracil potassium). Our patient died due to liver failure a month after initial admission. A pathological review of necropsy specimens of his liver lesions showed moderately differentiated tubular adenocarcinoma (Figure 4A). An immunohistochemical study showed that CEA-positive and AFP-negative cells were present in the metastatic liver tumor resembling the gastric lesion (Figures 4B and 4C).

## Discussion

NF-1, or von Recklinghausen's disease, was first described in 1882. It is a relatively common autosomal hereditary dominant disorder that equally involves men and woman and has variable expression, a frequency of one per 3000 births, and a high rate of new mutations [4]. The genetic basis of the disease is a mutation located on chromosome 17q11.2 [1]. NF-1 is characterized by brown skin pigmentation ('café au lait' spots), cutaneous neurofibromas and neoplasms of the peripheral or central nervous system. The association between NF-1 and tumors of neurogenic and neuroendocrine origin, such as meningiomas, gliomas and pheochromocytomas, is well known. Malignancies are found in 3% to 5% of patients. GI involvement is present in approximately a quarter of cases [2]. GI involvement in NF-1 commonly occurs in four principal forms: hyperplasia of the gut neural tissue, multiple GI stromal tumors (GISTs), duodenal or periampullary endocrine tumors and a miscellaneous group of other tumors. GISTs are the most common of these forms [1]. Although there have been scattered reports of adenocarcinoma of the GI tract complicating peripheral neurofibromatosis [1,3], few cases have explored the association of primary infiltrating adenocarcinoma of the stomach [5]. It has been indicated that loss of function of the *NF-1 *gene results in peripheral neurofibromatosis [6], but no definite reasons have been cited for the high incidence of primary malignant tumors in these patients. The *NF-1 *gene and the *p53 *tumor suppressor gene are both located on chromosome 17 (6), suggesting that the *p53 *gene is mutated in several NF-1-associated tumors [7]. Researchers have sought to determine the role of *p53 *tumor suppressor genes in the etiopathogenesis of NF-1-associated malignant primary tumors. In a recent study, a germline *NF-1 *nonsense mutation in exon 37 was detected by DNA sequence analysis, showing that the GI tumor arose through *NF-1 *gene inactivation [8]. The *NF-1 *gene product, neurofibromin, contains a guanosine 5'-triphosphate (GPT)ase-activating protein-related domain that is able to down-regulate p21ras by stimulating its intrinsic GPTase. Because p21ras-GPT is a major regulator of growth and differentiation, mutant neurofibromins resulting from somatic mutations in the *NF-1 *gene might interfere with ras signaling pathways and contribute to the development of tumors [9]. These results suggest a causal association between NF-1 and the development of gastric cancer in our case.

AFP- positive cases have been found among disorders other than hepatocellular carcinoma, such as hepatitis, liver cirrhosis and metastatic cancer of the liver. Additionally, elevated levels of serum AFP have been reported to occur with several tumor types other than hepatocellular carcinoma and embryonic cell carcinoma [10]. These elevated levels have largely been associated with neoplasms of the GI tract [11]. In the GI tract, an elevation of the serum AFP level was reported in 1.3% to 15% of gastric cancers [10]. The authors described AFP-producing gastric adenocarcinomas with a high serum AFP level and synchronous hepatic metastasis. Gastric cancers that secrete AFP are rare. The first case was described in 1970 [11]. AFP-secreting gastric cancers occur with a frequency of 2% to 6%. The prognosis of these cases tends to be poor with a high frequency of hepatic metastasis at presentation. Liver metastasis has been reported to occur in 70% to 80% of cases. Our patient had an elevated serum AFP level and a gastric cancer with liver metastasis without viral hepatitis or liver cirrhosis; however, the immunohistochemical analysis showed that the tumor was AFP-negative. Previous authors reported the occurrence of gastric adenocarcinoma with a high serum AFP level and synchronous hepatic metastasis [12]. It has subsequently become clear that there are two distinct histologic subtypes: a medullary type and a papillary or tubular type. The medullary type tends to stain more strongly for AFP [12]. Our case showed moderately differentiated tubular adenocarcinoma. Additionally, an immunohistochemical analysis showed that the cells present in the metastatic liver tumor and the gastric lesion were AFP-negative. The regulation of gastric cancer cell lines by hepatocyte growth factor (HGF) and *c-metproto-*oncogene *(c-Met*) has been described recently [13]. HGF is strongly associated with the progression of cancer cells to invasive phenotypes and the development of distant metastases. A higher incidence of *c-Met *overexpression was found in AFP-secreting tumors, as well as a higher expression in poorly differentiated tumors in the AFP-positive group than in those that were AFP-negative [13]. A serum AFP level over 500 ng/mL in gastric cancer is rare.

Studies have correlated levels of CA 72-4 with findings of pathologic examinations in gastric carcinoma. These have shown significantly higher marker levels associated with gastric serosa invasion by the neoplasia and invasion of veins or lymphatic vessels into the gastric wall as well as lymph-nodal metastases [14]. A more advanced stage of gastric cancer (stage III and IV) results in higher serum levels of CA 72-4. Our patient had a normal CA 19-9 serum level. Elevated serum levels of CA 19-9 have been described in 25% to 48% of patients with gastric cancers, but these patients had multiple liver metastases. No correlation was found between serum CA 19-9 level and the stage of gastric cancer [15].

## Conclusion

We have reported a rare gastric cancer in a patient with NF-1 with high serum levels of multiple serum tumor markers.

## Consent

Written informed consent was obtained from the patient's relatives for publication of this case report and any accompanying images. Copies of the written consent are available for review by the Editor-in-Chief of this journal.

## Competing interests

The authors declare that they have no competing interests.

## Authors' contributions

KK, TK and KO conceived and designed the report, analyzed all the reports and drafted the manuscript. YK and KK drafted the manuscript and searched the literature. AN and NM performed surgery on the patient and participated in designing the report. YI, MT and HF participated in designing the report. All authors read and approved the final manuscript.
